# Correlation and Comparative Evaluation of MOCART and MOCART 2.0 for Assessing Cartilage Repair

**DOI:** 10.3390/medicina61040745

**Published:** 2025-04-18

**Authors:** Felix Conrad Oettl, Louis Leuthard, Moritz Brunner, Vincent A. Stadelmann, Stefan Preiss, Michael Leunig, Gian M. Salzmann, Jakob Hax

**Affiliations:** 1 Department of Hip and Knee Surgery, Schulthess Klinik, 8008 Zurich, Switzerlandstefan.preiss@kws.ch (S.P.); michael.leunig@kws.ch (M.L.); gian.salzmann@kws.ch (G.M.S.); jakob.hax@kws.ch (J.H.); 2 Hospital for Special Surgery, New York, NY 10021, USA; 3 Department of Research and Development, Schulthess Klinik, 8008 Zurich, Switzerland; louis.leuthard@kws.ch (L.L.); vincent.stadelmann@kws.ch (V.A.S.); 4 Faculty of Medicine, University of Zurich, 8091 Zurich, Switzerland

**Keywords:** cartilage, MOCART, patient-reported outcome measures, minced cartilage, AutoCart and level of evidence

## Abstract

*Background and Objectives*: Chondral and osteochondral lesions can lead to osteoarthritis if untreated, making accurate assessment of cartilage repair outcomes essential for optimizing treatment strategies. The objective of this study was to compare MOCART and MOCART 2.0 and to evaluate the clinical utility of both across different surgical cartilage repair techniques and various time points. *Material and Methods*: This study included 111 patients (age: 35 ± 10, 35% female) who underwent cartilage repair surgery of the knee between September 2015 and March 2022. A total of 188 postoperative magnetic resonance images were evaluated using MOCART and MOCART 2.0. The correlations between both scores, as well as to the change in Patient-Reported Outcome Measures (PROMs), were determined. *Results*: MOCART 2.0 scores (66 ± 13) were significantly higher than MOCART scores (58 ± 13, *p* < 0.001). Positive correlation was observed between scoring systems (r = 0.837, *p* < 0.001). There was no significant correlation between MOCART or MOCART 2.0 scores and the change in PROMs. Noticeably, there was a statistically significant correlation between both MOCART and MOCART 2.0 in the AutoCart subgroup across multiple timepoints for the change in PROMs. *Conclusions*: Based on radiographic–clinical outcome discordance, clinicians should not rely solely on MOCART or MOCART 2.0 scores when evaluating cartilage repair success but instead prioritize patient-reported functional improvements while using imaging as a complementary assessment tool.

## 1. Introduction

Chondral and osteochondral lesions refer to acute or chronic defects involving the articular cartilage and, in some cases, the underlying subchondral bone. Chondral lesions are confined to the cartilage surface, while osteochondral lesions affect both the cartilage and the subchondral bone. The prevalence of chondral lesions is substantial, with studies reporting cartilage abnormalities in 60–65% of arthroscopic procedures [1,2]. Untreated lesions cause higher mechanical stress on the surrounding intact cartilage [3]; have an influence on the subchondral bone [4], as well as on the intra-articular milieu with an increase in cytokine concentration [5]; and can thus lead to the premature onset of osteoarthritis (OA). There are several surgical techniques, like minced cartilage implantation (MCI), AutoCart (AC), autologous chondrocyte implantation (ACI), and microfracturing (MF), for cartilage repair. Clinical radiological follow-up assessments of such methods remain the subject of current research and are important for establishing effective treatment strategies for diverse cartilage defects.

While patient-reported outcome measures (PROMs) [6,7] are clinically appropriate to evaluate overall joint health and burden of disease, they neglect to report on the quality of the repair tissue. MRI has emerged as the preferred technique for evaluating the structural characteristics of cartilage lesions and monitoring the progression of repair tissue [8,9].

Advanced MRI techniques have significantly improved the assessment of cartilage repair, offering non-invasive alternatives to second-look arthroscopy. Quantitative MRI methods, including T2 mapping, T1rho, dGEMRIC (delayed gadolinium-enhanced MRI of cartilage), and diffusion-weighted imaging, provide insights into the biochemical composition and ultrastructure of repair tissue [9,10,11]. The Magnetic Resonance Observation of Cartilage Repair Tissue (MOCART) score was introduced in 2004 [12,13] and has been used in numerous trials since [14,15,16]. With advancements in understanding of the osteochondral unit’s biology and progress in MRI techniques [17,18], the role of the subchondral bone in the process of cartilage repair was recognized, eventually leading to the development of the MOCART 2.0 [19]. The MOCART 2.0 reworked most variables, enabling a more granular assessment, eliminating the need for multiple MRI sequences, and increasing the emphasis of the subchondral bone [12,19].

This study aimed to compare MOCART and MOCART 2.0 scores following various cartilage repair techniques (MCI, AC, ACI, and MF), with follow-up periods ranging from 6 months to 5 years, and to evaluate their correlation with patient-reported outcome measures (PROMs). We hypothesize that MOCART and MOCART 2.0 scores will demonstrate a strong correlation, with MOCART 2.0 yielding significantly higher values, while both will show poor correlation with clinical outcomes. These findings will contribute to the evidence base guiding clinical decision-making in cartilage repair by clarifying relationships between surgical techniques, structural repair assessment methods, and patient-reported function.

## 2. Methods and Materials

### 2.1. Study Design

This single-center retrospective study used data extracted from our clinical information system (CIS) and from our institutional registry to evaluate and compare the efficacy of the MOCART and MOCART 2.0 scoring systems in assessing cartilage repair on postoperative MRI scans. The correlation between these MRI scoring systems and changes in PROMs from baseline was examined.

Ethical approval for our registry on lower extremity disorders, including the validation of assessment instruments, was obtained from the local ethics committee of the Canton of Zurich (KEK-ZH 2015-0258) on 23 June 2015. Written informed consent was obtained from all participants prior to data collection, and all data analyzed were completed in line with the 1964 Helsinki Declaration and its amendments.

### 2.2. Patient Selection

A total of 111 patients aged 18 to 80 years who underwent open or arthroscopic cartilage repair surgery for chondral and/or osteochondral lesions involving the femoral condyles, tibial plateau, trochlea, or patella of the knee joint between 2015 and 2022 at our institution were included. The eligible surgical procedures were MCI, ACI, AC, and MF. Traumatic cartilage lesions and those resulting from osteochondritis dissecans were included. Patients who underwent concomitant cartilage repair procedures at multiple sites within the same knee joint were also included, and the largest defect was evaluated. The availability of at least one postoperative MRI scan was mandatory for inclusion in the study.

### 2.3. Surgical Techniques

Prior to surgery, all patients underwent preoperative MRI and standard knee radiographs for diagnostic evaluation and surgical planning. The definitive indication for performing cartilage repair surgery was established upon standard arthroscopic examination of the knee. Intraoperative grading of the cartilage defect was conducted in accordance with the International Cartilage Repair Society (ICRS) classification guidelines [20].

Chondral and osteochondral lesions were treated with either an open or arthroscopic approach utilizing MCI, AC, MF, or two-stage ACI, respectively [21,22,23,24]. In all cartilage repair procedures mentioned, the defects were first debrided and prepared to achieve stable and healthy cartilage margins. This was performed arthroscopically or open.

### 2.4. Patient-Reported Outcomes Collection

PROMs were extracted from the institutional registry, in which PROMs were prospectively collected preoperatively and at 6, 12, 24, and 60 months postoperatively. The questionnaires were sent out by post or e-mail and filled out by the patients at home. The following instruments were included: the IKDC [25] score about symptoms, sport activities, and knee function ranging from 0 (worst) to 100 (best), as well as the COMI [26] knee score, a short multidimensional (pain, function, disability, quality of life) questionnaire ranging between 0 (best) and 10 (worst). For correlation analysis, both IKDC and COMI were analyzed as changes from baseline (cIKDC, cCOMI)

### 2.5. Radiographic Data Collection and Assessment

Preoperative 3-T MR images of the knee were assessed from multiple independent scanners. A trained investigator blinded to surgical outcomes evaluated the MRIs using the Area Measurement and Depth and Underlying Structures (AMADEUS) scoring system [27] to quantify the severity of chondral and osteochondral defects prior to cartilage repair procedures. Postoperative MRIs are not established as standard of care and therefore were performed on various MRI scanners in and out of the center as required on a patient-by-patient basis. The indication for a postoperative MRI was given based on clinical presentation, especially in patients with persistent symptoms. For the evaluation of the repair tissue, the original MOCART [12,13] and the recently published MOCART 2.0 [19] were utilized on the full range of 0 (worst) to 100 (best) points. MRIs were analyzed in a PACS client (JiveX Version 5.3.0.7, Visus Health IT GmbH, Bochum, Germany).

Compared to the original MOCART system, MOCART 2.0 introduced several modifications. While the original MOCART [12,13] included 9 variables, the MOCART 2.0 [19] was reduced to 7 variables, with adjustments made to 4 of the remaining variables (Table 1).

### 2.6. Statistical Analysis

Statistical analysis was performed in RStudio using R version 4.3.1 (Vienna, Austria: R Foundation for Statistical Computing. 2021). Shapiro–Wilk was used to test the dataset for normality. Continuous variables demonstrating normality were compared between groups by independent Student *t*-tests. Pearson’s correlation coefficient assessed linear relationships between MOCART and MOCART 2.0 scores with time since surgery and changes from baseline in COMI and IKDC. MRI scans were matched with the chronologically closest PROMs available, with a maximum of two months between the MRI acquisition and PROM collection. Continuous variables were presented as mean ± standard deviation, and *p*-value < 0.05 was considered statistically significant for all assessments. A post hoc power analysis was performed for all statistically significant results using Gpower (3.1.9.7).

## 3. Results

Of the included 111 patients, a total of 180 MRIs with assignable PROMs were available and were used for analysis. The mean follow-up was 15.3 ± 14.0 months after surgery. Patient properties and lesion characteristics are presented in Table 2.

### 3.1. Radiographic Outcomes

MOCART and MOCART 2.0 showed a strong positive correlation (r = 0.84, *p* < 0.001), with all subgroups showing moderate to strong correlations. Post hoc power analysis showed a power of 1 for all subscores and the total score (Table 3).

### 3.2. Relationship Between MOCART/MOCART 2.0 and Time of MRI

The correlation between time since surgery and radiographic score was neither significant for MOCART (r = 0.14, *p* = 0.05) nor MOCART 2.0 (r = 0.06, *p* = 0.4). However, the signal intensity of repair tissue subscores of both MOCART and MOCART 2.0 did show a significant increase over time (r = 0.17, *p* = 0.02 and r = 0.15, *p* = 0.04, respectively).

### 3.3. Relationship Between MOCART/MOCART 2.0 and PROMs

COMI and IKDC scores showed improvement from baseline throughout the follow-up period. No associations between MOCART or MOCART 2.0 scores and changes in PROMs were observed (cCOMI; r = 0.06, *p* = 0.442; r = 0.08, *p* = 0.303; cIKDC; r = 0.06, *p* = 0.477; r = 0.08, *p* = 0.3293) when pooling techniques and timepoints. In the AC subgroup, a statistically significant correlation was observed between both MOCART and MOCART 2.0 scores, as well as cCOMI and cIKDC at multiple postoperative timepoints. Significant correlation persisted in the combined timepoint analysis (cCOMI: MOCART r = 0.45, *p* = 0.0197; MOCART 2.0 r = 0.43, *p* = 0.027; cIKDC: MOCART r = 0.6, *p* = 0.0015; MOCART 2.0 r = 0.73, *p* < 0.001). Furthermore, the MCI subgroup showed statistically significant correlations for cIKDC at the 24-month timepoint with MOCART and at the 12-month timepoint for MOCART 2.0 (r = 1, *p* < 0.001; r = 0.67, *p* = 0.0173); however these findings were based on small subgroups (n = 5 and n = 12, respectively). All other subgroups failed to show a statistically significant correlation between MOCART scores and PROMS (Table 4 and Table 5).

For MOCART, the MCI subgroup at 24 months showed a strong correlation with cIKDC (r = 1.00, power = 1, n = 5), while the AC subgroup demonstrated significant correlations with both cCOMI and cIKDC at 12 months (r = 0.64 and 0.65, power = 0.614 and 0.634, n = 11) and at 60 months (r = 0.45 and 0.60, power = 0.659 and 0.913, n = 25). Similarly, for MOCART 2.0, the AC subgroup at 12 and 24 months showed strong correlations (r = 0.72–0.80, power = 0.657–0.912, n = 9–11), as well as at 60 months (r = 0.73, power = 0.993, n = 25).

Associations between the clinical outcomes, denoted as cCOMI and cIKDC scores, and the individual subscores of the MOCART and MOCART 2.0 scoring systems were also evaluated. Numerous subscores across surgical methods and timepoints exhibited statistically significant correlation. Integration into the border and integration into adjacent cartilage most consistently showed a greater decrease in COMI and increase in IKDC across various timepoints and methodologies.

## 4. Discussion

The key findings of this study are (i) the strong correlation of the MOCART 2.0 with the original MOCART, (ii) the significantly higher MOCART 2.0 score compared to the original MOCART score, (iii) statistically significant association between MOCART scores and PROMs in the AC subgroup but not in the other techniques, and (iv) the MOCART 2.0 not showing superior correlation with clinical outcomes.

The clinical relevance of MRI scores, particularly the MOCART score, has been debated ever since their introduction [12,28,29,30]. Large discrepancies have been shown in the range of correlation coefficients, as well as their statistical significance, ranging from poor correlation without statistical significance to excellent correlation with high statistical significance, not only in composite scores but also in subcategories of the respective scores, which is in line with what we found for the majority of subgroups in our analysis [12,28,31,32]. This heterogeneity of the MRI score correlation with clinical outcomes is continued in the MOCART 2.0 [33,34,35]. Significant correlations between PROMS and both MOCART scores in the AC and MCI subgroups, not in the MF and ACI subgroups, confirming the heterogeneous results of others, were found. Conversely, correlation with the respective subscores showed high levels of correlation, especially with border integration metrics. It has to be noted that the PROMs in patients undergoing procedures which do not, in general, affect the subchondral bone (AC, MCI) correlated more strongly with both MOCART scores.

Our findings of inconsistent correlations between imaging metrics and clinical outcomes are further corroborated by recent literature. A 2023 investigation of autologous minced cartilage repair reported moderate MOCART 2.0 scores (62.3 ± 17.4) alongside satisfactory clinical outcomes yet demonstrated similar limitations in establishing clear correlations between radiographic and functional results, mirroring the heterogeneity we observed, particularly in the MF and ACI subgroups [36]. This pattern of disconnect between imaging and clinical measures was reinforced by a 2021 systematic review finding no statistically significant associations between MOCART scores and established tools, like IKDC, across knee and talus repairs [37]. In contrast, and highlighting the complexity we encountered, a 2024 study on patellofemoral cartilage repair found that higher MOCART scores correlated significantly with PASS achievement (*p* = 0.011), though it failed to demonstrate relationships with MCID for KOOS QoL [38]. These mixed results from contemporary research reinforce our observation that MRI scoring systems correlate differently across repair techniques, with our AC subgroup showing stronger correlations than other techniques. Such variability suggests that current radiographic assessment tools, while valuable, remain insufficient as standalone outcome predictors, especially in the clinically symptomatic population we examined, where imaging findings must be interpreted within the broader clinical context.

Schreiner et al. [19] compared the ICCs using the MOCART 2.0 of two experienced examiners (ICC 0.84) with that of an experienced versus an inexperienced examiner, who only had the MOCART atlas as a guide (ICC 0.57). Thus, the MOCART 2.0 requires a certain level of experience to correctly read the MRI images and should therefore remain in the hands of experienced examiners.

MOCART score changes over time have been shown to be heterogeneously dispersed in the body of literature, which is also discussed as a cause for the lack of correlation between MRI scores and clinical outcomes [39]. When evaluating such results, one, however, has to account for the limitation of such studies being underpowered, as is ours, as there are limited patients receiving postoperative MRIs. While the process and timeframe of graft maturation, and therefore morphology, is well established, resulting in increased MRI scores in early follow-up, the literature on long-term graft quality is heterogenous. Prospective or retrospectively matched studies in a diverse patient population are required to formulate a more definitive answer [12,39,40,41]. A significant increase in the MOCART score was found over time. Furthermore, significant positive correlations of the “Signal intensity of repair tissue” subscore in both scoring systems were detectable.

Systematic reviews have failed to identify associations between MRI metrics and clinical outcomes following cartilage repair procedures [28,29]. De Windt et al. assessed 32 investigations, concluding that more sophisticated imaging techniques might be a requisite for reliable post-procedural outcome prognostication in this population [28]. The lack of correlation between MOCART parameters and clinical outcome measures may also relate to the precise post-surgical interval at which MRI assessment is performed, as well as the lack of power in respective studies [40,41].

Literature assessing the correlation between MOCART 2.0 and clinical outcomes remains sparse due to its novelty. Casari et al. investigated the relationship between PROMs and MOCART and MOCART 2.0 in AMIC-treated osteochondral lesions of the talus, failing to find a significant correlation between radiographic and clinical scores [39]. Goller et. al., however, found a significant correlation between MOCART 2.0 and Delta IKDC between multiple subsets and timepoints in their analysis of retropatellar matrix-associated chondrocyte transplantation [34].

The current study exclusively included patients with unplanned follow-up MRI examinations conducted within 5 years post-operatively for patients presenting with recent trauma, pain, or undergoing revision surgery. Consequently, lower radiographic scores and PROMs were expected to be relative to previously published data [42,43,44]. Given that follow-up MRI scans in this analysis were only conducted for clinical indications, it is reasonable to hypothesize that higher average radiographic scores, as well as PROMs, may have been attained if data from the entire cohort of patients undergoing cartilage repair surgery were analyzed, irrespective of clinical presentation. This restriction to symptomatic patients narrows the range of observed data, thereby limiting the study’s ability to accurately assess the true correlation across the full spectrum of investigated variables.

The primary limitation to be noted is the heterogeneity in cartilage repair techniques used, which themselves evolved over time, possibly disguising significant correlations. Due to the retrospective nature of the study, the exact timepoint of MRI can vary and lead to a lack of consistency in the MRI scanner used across the patient cohort. The radiological measurements were performed by only one experienced examiner, though the quality of the data evaluation can be rated as high. Lastly, despite the more realistic scenario, the heterogeneity of non-cartilage injuries, as well as concomitant non-cartilage repair procedures performed simultaneously in our patient population, has to be seen as a limitation. Randomized controlled trials with homogenous patient populations and isolated cartilage injuries, as well as isolated cartilage repairs, would be required to evaluate the importance of morphological assessment on clinical outcomes more precisely.

## 5. Conclusions

The MOCART 2.0 score, while yielding significantly higher absolute values, does not demonstrate superior predictive capability for clinical outcomes across various cartilage repair techniques. Based on these findings, we recommend reporting both MOCART and MOCART 2.0 scores in cartilage repair studies to maintain comparability across the literature, despite the additional workload this entails. Further investigation of the MOCART 2.0 is warranted, particularly through prospective comparative studies with the original MOCART, to fully establish its clinical utility and validate its enhanced assessment parameters.

## Figures and Tables

**Table 1:** Comparison of the compositions of MOCART and MOCART 2.0.

Variables (MOCART/MOCART2.0 if Diverse)	MOCART [12,13]	MOCART 2.0 [19]
Degree of defect repair and filling of the defect/volume fill of the cartilage defect	20	20
Integration to border zone/integration into adjacent cartilage	15	15
Surface of the repair tissue	10	10
Structure of the repair tissue	5	10
Signal intensity of the repair tissue	30	15
Subchondral lamina	5	-
Subchondral bone	5	-
Adhesions	5	-
Effusion	5	-
Bony defect or bony overgrowth	-	10
Subchondral changes	-	20
TOTAL	100	100

MOCART Magnetic Resonance Observation of Cartilage Repair Tissue.

**Table 2:** Patient properties and lesion characteristics.

Characteristic	N = 111 ^1^	ACI, N = 36 ^1^	AC, N = 16 ^1^	MCI, N = 22 ^1^	MF, N = 37 ^1^	*p*-Value
Age [years]	35 ± 10	34 ± 9	36 ± 13	33 ± 10	38 ± 9	0.205
BMI [kg/m^2^]	24.7 ± 3.6	23.8 ± 3.3	24.7 ± 4.3	23.9 ± 3.0	26.0 ± 3.8	0.048
Weight [kg]	78 ± 14	76 ± 14	77 ± 13	75 ± 12	82 ± 14	0.166
Sex						0.800
Male	72 (65%)	21 (58%)	11 (69%)	15 (68%)	25 (68%)	
Female	39 (35%)	15 (42%)	5 (31%)	7 (32%)	12 (32%)	
ASA Class						0.948
1	71 (64%)	24 (67%)	10 (63%)	13 (59%)	24 (65%)	
2	39 (35%)	12 (33%)	6 (38%)	9 (41%)	12 (32%)	
3	1 (0.9%)	0 (0%)	0 (0%)	0 (0%)	1 (2.7%)	
Symptom duration [d]	21 ± 27	28 ± 34	17 ± 20	30 ± 33	11 ± 12	0.004
Initial traumatic event	69 (63%)	27 (75%)	12 (75%)	7 (33%)	23 (62%)	0.011
Defect size [cm^2^]	3.24 ± 2.01	4.38 ± 2.06	2.91 ± 1.34	4.03 ± 2.02	1.81 ± 1.13	<0.001
ICRS grade						0.556
3	85 (86%)	30 (86%)	11 (79%)	13 (81%)	31 (91%)	
4	14 (14%)	5 (14%)	3 (21%)	3 (19%)	3 (8.8%)	
Defect location						
Patella	35 (32%)	21 (58%)	2 (13%)	9 (41%)	3 (8.1%)	<0.001
Trochlea	31 (28%)	12 (33%)	5 (31%)	2 (9.1%)	12 (32%)	0.149
Med. Femoral condyle	45 (41%)	9 (25%)	9 (56%)	7 (32%)	20 (54%)	0.033
Lat. Femoral condyle	10 (9.0%)	2 (5.6%)	2 (13%)	3 (14%)	3 (8.1%)	0.642
Other	2 (1.8%)	0 (0%)	0 (0%)	0 (0%)	2 (5.4%)	>0.999
AMADEUS Score	58 ± 16	56 ± 16	60 ± 12	44 ± 17	67 ± 10	<0.001
COMI	5.22 ± 1.69	5.14 ± 1.59	5.43 ± 1.48	5.26 ± 1.62	5.18 ± 1.95	0.928
IKDC	48 ± 14	47 ± 14	47 ± 16	51 ± 12	48 ± 14	0.666

^1^ Mean ± SD; N (%), AMADEUS score averages calculated across defect locations. COMI: Core Outcome Measures Index, IKDC: International Knee Documentation Committee, AMADEUS: Area Measurement and Depth and Underlying Structures, ICRS: International Cartilage Repair Society.

**Table 3:** Comparison of MOCART and MOCART 2.0 and their correlation.

MOCART	Mean ± SD	MOCART 2.0	Mean ± SD	*p*	r-Correlation	*p*-Correlation
Degree of defect repair and defect filling	14.8 ± 4.7	Volume of cartilage defect filling compared to native cartilage	15.2 ± 4.7	0.255	0.94	<0.001
Integration to border	10.1 ± 2.8	Integration into adjacent cartilage	10.3 ± 2.6	0.377	0.92	<0.001
Surface of the repair tissue	5.9 ± 2.8	Surface	5.9 ± 2.7	0.873	0.98	<0.001
Structure of the repair tissue	3.46 ± 2.32	Structure	7 ± 4.6	<0.001	0.98	<0.001
Signal intensity of the repair tissue	11 ± 6	Signal intensity of the repair tissue	9.95 ± 2.64	0.419	0.78	<0.001
Subchondral lamina	1.52 ± 2.3	Bony defect or bony overgrowth	5.6 ± 3.0	<0.001	0.71	<0.001
Subchondral bone	1.36 ± 2.23	Subchondral changes	12 ± 6	<0.001	0.77	<0.001
Adhesions	4.52 ± 1.48	-	-	-		
Effusion	4.65 ± 1.27	-	-	-		
Total MOCART	58 ± 13	Total MOCART 2.0	66 ± 13	<0.001	0.84	<0.001

MOCART: Magnetic Resonance Observation of Cartilage Repair Tissue, r: Pearson correlation coefficient, *p*: significance.

**Table 4:** Subgroup correlations between MOCART and PROMs.

MOCART	6 Months		12 Months		24 Months		60 Months		Combined	
	cCOMI	cIKDC	cCOMI	cIKDC	cCOMI	cIKDC	cCOMI	cIKDC	cCOMI	cIKDC
MF	0.15	0.18	0.12	0.37	0.09	0.27	0.2	0.44	0.06	0.04
MCI	0.33	0.39	0.5	0.53	0.54	**1**	NA	NA	0.18	0
AC	0.29	0.65	**0.64**	**0.65**	0.08	0.22	NA	NA	**0.45**	**0.6**
ACI	0.2	0.11	0.21	0.16	0.01	0.06	NA	NA	0.03	0.1
Combined	0.03	0.01	0.13	0.11	0.13	0.2	0.23	0.15	0.06	0.06

Data displayed as Pearson correlation coefficient (r). In the presence of statistically significant correlation, r is printed in bold (*p* < 0.05).

**Table 5:** Subgroup correlations between MOCART 2.0 and PROMs.

MOCART 2.0	6 Months		12 Months		24 Months		60 Months		Combined		
	cCOMI	cIKDC	cCOMI	cIKDC	cCOMI	cIKDC	cCOMI	cIKDC	cCOMI	cIKDC	
MF	0.03	0.03	0.31	**0.62**	0.02	0.09	0.07	0.43	0.08	0.15	
MCI	0.16	0.07	0.05	**0.67**	0.56	0.66	NA	NA	0.05	0.23	
AC	0.1	**0.72**	**0.72**	**0.8**	0	0.43	NA	NA	**0.43**	**0.73**	
ACI	0.15	0.02	0.16	0.04	0.05	0.14	NA	NA	0.03	0.06	
Combined	0.02	0.06	0.24	0.14	0.05	0.09	0.04	0.2	0.08	0.08	

Data are displayed as Pearson correlation coefficients (r). In the presence of statistically significant correlation, r is printed in bold (*p* < 0.05).

## Data Availability

The data presented in this study are available on reasonable request from the corresponding author.

## Abbreviations

COMI	Core Outcome Measures and Index
AC	AutoCart
ACI	Autologous Chondrocyte Implantation
AMADEUS	Area Measurement and Depth and Underlying Structures
cCOMI	Change in Core Outcome Measures and Index
cIKDC	Change in Knee Documentation Committee
CIS	Clinical Information System
ICRS	International Cartilage Repair Society
MCI	Minced Cartilage Implantation
MF	Microfracturing
MOCART	The Magnetic Resonance Observation of Cartilage Repair Tissue
OA	Osteoarthritis
PROMs	Patient-Reported Outcome Measures
